# The rate of TB-HIV co-infection depends on the prevalence of HIV infection in a community

**DOI:** 10.1186/1471-2458-8-266

**Published:** 2008-07-30

**Authors:** Daniel G Datiko, Mohammed A Yassin, Luelseged T Chekol, Lopisso E Kabeto, Bernt Lindtjørn

**Affiliations:** 1 Southern Nations, Nationalities and Peoples' Regional Health Bureau, P.O. Box 149, Awassa, Ethiopia; 2 Liverpool School of Tropical Medicine, Pembroke place, L3 5QA, Liverpool, UK; 3 Centre for International Health, University of Bergen, Armauer Hansen Building, N-5012, Bergen, Norway

## Abstract

**Background:**

A complex interaction exists between tuberculosis (TB) and human immunodeficiency virus (HIV) infection at an individual and community level. Limited knowledge about the rate of HIV infection in TB patients and the general population compromises the planning, resource allocation and prevention and control activities. The aim of this study was to determine the rate of HIV infection in TB patients and its correlation with the rate HIV infection in pregnant women attending antenatal care (ANC) in Southern Ethiopia.

**Methods:**

All TB patients and pregnant women attending health institutions for TB diagnosis and treatment and ANC were consecutively enrolled in 2004 – 2005. TB diagnosis, treatment and HIV testing were done according to the national guidelines. Blood samples were collected for anonymous HIV testing. We used univariate and multivariate logistic regression analysis to determine the risk factors for HIV infection and linear regression analysis to determine the correlation between HIV infection in TB patients and pregnant women.

**Results:**

Of the 1308 TB patients enrolled, 226 (18%) (95%CI: 15.8 – 20.0) were HIV positive. The rate of HIV infection was higher in TB patients from urban 25% (73/298) than rural areas 16% (149/945) [AOR = 1.78, 95%CI: 1.27–2.48]. Of the 4199 pregnant women attending ANC, 155 (3.8%) [95%CI: 3.2–4.4] were HIV positive. The rate of HIV infection was higher in pregnant women from urban (7.5%) (80/1066) than rural areas (2.5%) (75/3025) [OR = 3.19, 95% CI: 2.31–4.41]. In the study participants attending the same health institutions, the rate of HIV infection in pregnant women correlated with the rate of HIV infection in TB patients (R^2 ^= 0.732).

**Conclusion:**

The rate of HIV infection in TB patients and pregnant women was higher in study participants from urban areas. The rate of HIV infection in TB patients was associated with the prevalence of HIV infection in pregnant women attending ANC.

## Background

The interaction between tuberculosis (TB) and human immunodeficiency virus (HIV) infection is complex. In the individual patient, HIV infection weakens the immune system and increases the susceptibility to TB. HIV increases the likelihood of reactivation, reinfection and progression of latent TB infection to active disease. It also alters the clinical presentation of TB, complicates the follow up and compromises the response to anti-TB treatment [1].

In a population, the lifetime risk of developing active TB once infected, in absence of HIV infection, is about 10% [2]. However, it increases tenfold in HIV infected individuals. This has resulted in a large increase in the number of TB cases [3,4]. The proportion of smear-negative pulmonary TB (PTB) and extrapulmonary TB (EPTB) is higher among HIV co-infected TB patients [5].

At TB control programme level, an increase in the TB burden leads to increased need of trained staff, diagnostic facilities and patient care. The number of smear positive PTB cases registered has been used as the basis for procurement and distribution of drugs and supplies [6]. However, changes in the proportion of smear negative PTB and EPTB due to HIV co-infection may require adjustments. In Ethiopia, ten per cent of HIV infected people require antiretroviral therapy and the need is more among TB patients co-infected with HIV [7]. Therefore, knowledge about the rate of HIV infection in TB patients might help in planning and resource allocation. Regular surveillance of HIV infection in TB patients and the general population would also help in understanding the spread of the dual infections and monitoring the performances of TB and HIV control activities [8,9].

However, knowledge about the prevalence of HIV infection in the general population and its correlation with the rate of HIV infection in TB patients is limited in Ethiopia. The aim of this study was to determine the rate of HIV infection in TB patients and its correlation with the rate HIV infection in pregnant women attending antenatal care (ANC) in Southern Ethiopia.

## Methods

### Study area and population

This study was conducted in the Southern Nations, Nationalities and Peoples' Region (SNNPR) of Ethiopia. The region has 13 administrative zones and an estimated population of 14 million, of which 93% live in rural areas. Only half of the population live within two-hour walking distance from a public health institution. The Regional Health Bureau has adopted the World Health Organization recommended directly observed short course treatment strategy for TB control since 1995. The first round HIV survey among TB patients and pregnant women was conducted in 2002 [10]. In this study, the number of surveillance sites was increased to include more urban and rural communities to represent all zones of the region.

### Study design and site selection

This is a cross-sectional study carried out from September 2004 to April 2005.

### TB-HIV co-infection survey

**Figure 1:**
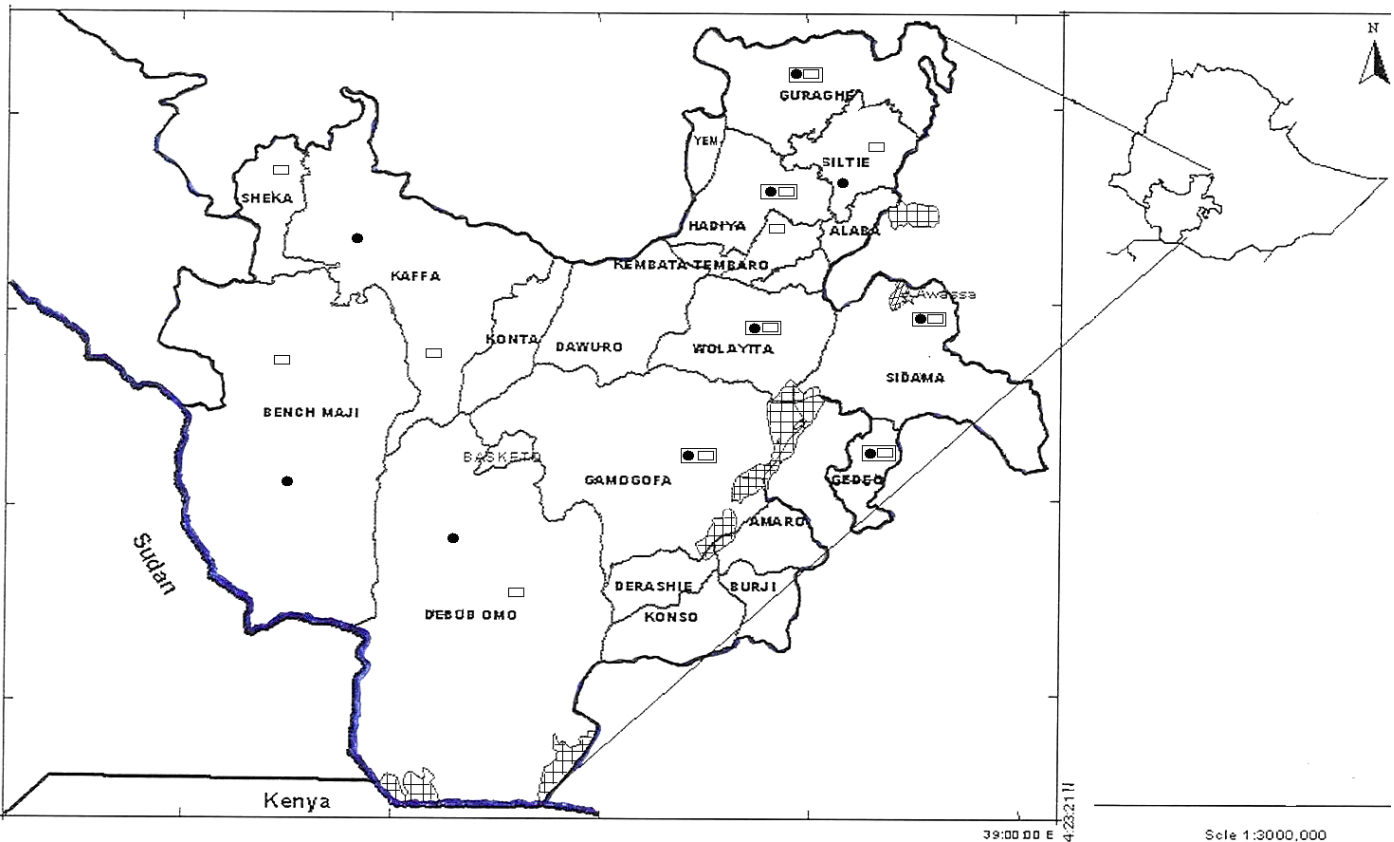
**Map of the Southern Nations, Nationalities and Peoples' Region of Ethiopia showing the survey sites, 2004 – 2005**.  Zonal boundary.  Regional boundary.  International boundary.  Regional capital, Awassa.  Lake.  TB-HIV survey sites.  ANC-based sentinel survey sites.  Overlapping sites.

Health institutions were selected based on their capacity to diagnose and treat TB patients. The diagnostic services included direct sputum microscopy, routine blood tests and x-rays. Ten health institutions (Figure 1) were randomly selected. All TB patients were consecutively enrolled at their first visit to the treatment units.

### ANC – based HIV sentinel survey

Health institutions that deliver ANC, had an adequate client volume, collect blood samples for routine tests such as haemoglobin determination and syphilis testing and facilities to maintain cold chain were identified of which twelve health institutions (Figure 1) were randomly selected. All pregnant women attending ANC were consecutively enrolled at their visit to health institutions [11].

In both surveys, TB patients and pregnant women referred from other health institutions or coming for the second visit during the survey period were excluded to avoid repetition. In six of the study sites, both surveys were conducted in the same health institutions providing health service to TB patients and pregnant women from the same districts. However, in the remaining sites, the surveys were conducted in health institutions providing health service to the population in the nearby districts.

### Diagnosis of TB

The diagnosis of TB was based on the recommendations of the National TB and Leprosy Control Programme [6]. Briefly, patients presenting with symptoms suggestive of PTB who had productive cough for three weeks or more with at least two positive sputum smears or one positive smear and x-ray findings consistent with active PTB were classified as smear-positive PTB cases. Patients presenting with cough of three weeks or more with initial three negative smears and no clinical response to a course of broad-spectrum antibiotics, three negative smear results after a course of broad-spectrum antibiotics, x-ray findings consistent with active PTB and decided by a clinician to be treated with anti-TB chemotherapy were classified as smear-negative PTB cases. Patients presenting with dry cough of three weeks or more were diagnosed based on strong clinical evidence and x-ray findings consistent with active TB. Patients presenting with symptoms suggestive of TB other than the lungs, which did not respond to a course of broad-spectrum antibiotics and decided by a clinician to be treated with anti-TB chemotherapy were classified as EPTB cases. In children, TB was diagnosed if there were symptoms and signs suggestive of TB, contact history with a known TB patient and x-ray findings consistent with active TB.

### Data and specimen collection

Trained laboratory technicians and health workers from TB and ANC functions collected the data using pretested questionnaires. The main variables were age, sex, residence and survey site for all participants, and disease classification and category for TB patients. 5 ml of blood samples were collected from TB patients and pregnant women. Routine blood tests except for HIV were done locally and reported to the attending health workers. The remaining serum samples were stripped off individual identifying markers and were assigned unique codes. They were kept at 4°C, transported to the regional Centre for Health Research Laboratory (CHRL) and stored at – 20°C until analysis. The serum samples were anonymously tested for HIV using ELISA test (Vironostica ^® ^Uniform II Ag/Ab BIOMÉRIEUX). All the samples were sent to the Ethiopian Health and Nutrition Research Institute (EHNRI) to repeat ELISA test using Enzygnost Anti-HIV1/2 Plus (Dade Behring, Germany) and quality control. ELISA reactive specimens at CHRL and EHNRI were considered positive and discordant specimens were retested using similar tests [11,12].

### Data analysis

We used SPSS 14.0 (SPSS Inc, Chicago, IL, USA) for data entry and analysis. We determined the rate of HIV infection in TB patients and pregnant women. Univariate and multivariate logistic regression analysis were used to determine the risk factors for HIV infection in TB patients and pregnant women. Socio-demographic variables that were significant by univariate analysis were included in the model to calculate adjusted odds ratio and 95% confidence interval by HIV status in TB patients. We also did linear regression analysis to determine the variation of HIV infection in TB patients explained by the prevalence of HIV infection among pregnant women from all study sites and then for the study participants from the same health institutions. P-value < 0.05 was considered as statistically significant.

### Ethical clearance

Ethical Review Committee of the Regional Health Bureau approved the study. Oral informed consent was obtained for all study participants. The study participants who wanted to know their HIV status were advised to go to voluntary counselling and testing service located within the health institutions or nearby.

## Results

1308 TB patients and 4199 pregnant women were included in the study. Of the TB patients, 729 (56%) were men and 569 (44%) were women. 309 (24%) patients came from urban and 978 (76%) patients from rural areas. Their mean age was 28.4 years. 544 (42%) patients had smear-positive PTB, 449 (34%) smear-negative PTB and 308 (24%) EPTB. The rate of HIV infection in TB patients was 18% (226/1261) [95%CI: 15.8–20.0] ranging from 8.3% (in Silte zone) to 35.3% (in South Omo zone). The rate of HIV infection in TB patients was similar for men and women (OR = 1.00, 95%CI: 0.75 – 1.34). There was no difference in the rate of HIV infection by TB disease classification: the rate of HIV infection among smear-positive PTB cases 17.5% (92/526) was similar to smear-negative PTB 18.1% (78/432) [OR = 1.048, 95%CI: 0.723–1.519] and EPTB cases 18.2% (54/297) [OR = 1.009, 95%CI: 0.687–1.480]. The rate of HIV infection was higher in TB patients from urban (24.5%, 73/298) than rural areas (15.8%, 149/945) [AOR = 1.78, 95%CI: 1.27–2.48] as shown in Table 1 &3.

**Table 1:** Socio-demographic characteristics and HIV status of TB patients, southern Ethiopia, 2004 – 2005

**Variables**		**TB Patients without HIV ** **(N = 1035), n (%)**	**TB patients with HIV ** **(N = 226), n (%)**	**OR (95%CI)**	**P-value**	**AOR (95%CI)**	**P-value**
Age	Mean (SD)	29.24 (9.85)	28.29 (13.77)				
Gender	Male	581(82.1)	127 (17.9)	1			
	Female	445 (82.1)	97 (17.9)	0.99 (0.75 – 1.34)	0.985		
Residence	Rural	796 (84.2)	149 (15.8)	1			
	Urban	225 (75.5)	73 (24.5)	1.73 (1.26 – 2.38)		1.77 (1.28 – 2.46)	0.001
Age group	0 – 14	109 (90.8)	11 (9.2)	1			
	15 – 24	344 (88.0)	47 (12.0)	1.35 (0.68 – 2.70)		2.01 (0.54 – 7.49)	0.301
	25 – 34	267 (73.4)	97 (26.6)	3.60 (1.86 – 6.98)		2.54 (0.76 – 8.46)	0.129
	35 – 44	153 (76.9)	46 (23.1)	2.98 (1.48 – 6.01)		7.10 (2.17 – 23.26)	0.001
	45 – 54	113 (86.9)	17 (13.1)	1.76 (0.78 – 3.93)		5.78 (1.72 – 19.38)	0.005
	≥ 55	57 (95.0)	3 (5.0)	0.52 (0.14 – 1.95)		3.34 (0.94 – 11.93)	0.063
TB classification	PTB +ve	434 (82.5)	92 (17.5)	1			
	PTB -ve	354 (81.9)	78 (18.1)	1.04 (0.75 – 1.45)	0.82		
	EPTB	243 (81.8)	54 (18.2)	1.05 (0.72 – 1.52)	0.803		
TB category	New	956 (82.6)	202 (17.4)	1			
	RFDO	32 (74.4)	11 (25.6)	1.61(0.79 – 3.24)	0.184		

TB = Tuberculosis, HIV = Human immunodeficiency virus, OR = odds ratio, CI = confidence interval, AOR = adjusted OR for age and residence

SD = standard deviation, PTB +ve = smear positive pulmonary TB, PTB -ve = smear negative pulmonary TB, EPTB = extrapulmonary TB, R = relapse,

F = failure, D = return after default, O = others. Missing variables: age – 15 (1.2%), sex – 12(0.9%), address – 21(1.6%), disease classification – 7(0.5%), disease category – 58(4.4%), HIV result – 47(3.6%), sex & HIV result – 58(4.4%), age group and HIV – 61(4.7%), address and HIV – 21(1.6%), disease classification and HIV – 53(4.1%) and age category and HIV- 61(4.7%).

Of the 4199 pregnant women attending ANC, 3097 (74%) came from rural and 1096 (26%) from urban areas. Their mean age was 25.7 years. The prevalence of HIV infection among the pregnant women was 3.8% (155/4091) [95%CI: 3.2–4.4] ranging from 1.5% (in Gamo Goffa zone) to 10.5% (in Wolaita zone). The rate of HIV infection was higher among women from urban 7.5% (80/1066) than rural 2.5% (75/3025) areas [OR = 3.19, 95% CI: 2.31–4.41] (Table 2 &3).

**Table 2:** Socio-demographic characteristics and HIV status of pregnant women attending ANC, Southern Ethiopia, 2004 – 2005

**Variables**		**ANC attendants without HIV ** **(N = 3936), n (%)**	**ANC attendants with HIV ** **(N = 155), n (%)**	**OR (95%CI)**	**P – value**
Age	Mean (SD)	25.45 (5.25)	25.72 (5.19)		
Age group	15 – 24	1547 (96)	64 (4.0)	1	
	25 – 34	2077 (96.4)	77 (3.6)	0.89 (0.64 – 1.26)	0.525
	≥ 35 – 44	312 (95.7)	14 (4.3)	1.09 (0.60 – 1. 96)	0.788
Residence	Rural	2950 (97.5)	75 (2.5)	1	
	Urban	986 (92.5)	80 (7.5)	3.19 (2.31 – 4.41)	0.0001

ANC = antenatal care, HIV = human immunodeficiency virus, OR = odds ratio, CI = confidence interval, SD = standard deviation

Missing variables: age – 6(0.1%), address – 6(0.1%), HIV result – 108(2.6%), age group & HIV – 108(2.6%), address & HIV – 108(2.6%)

**Table 3:** The rate of HIV infection among TB patients and pregnant women attending antenatal care in southern region of Ethiopia 2004 – 2005

Survey sites by zones*	**ANC attendants with HIV % (N)**	**TB patients with HIV % (N)**	**R^2†^**	**Adjusted R^2^**	**P-value^‡^**
**Urban survey sites**					
Sidama zone	9.48 (29/306)	17.84 (38/213)			
Wolaita zone	10.53 (26/247)	13.79 (12/87)			
Gedeo zone	9.46 (21/222)	18.11 (23/127)			
Bench Maji zone	2.25 (8/360)	32.5 (66/203)			
South Omo zone	1.72 (7/408)	35.29 (12/34)			
Kaffa zone	2.45 (8/326)	26.23 (16/61)			
**Rural survey sites**					
Hadiya zone	2.7 (7/259)	9.17 (21/229)			
Gurage zone	4.5 (18/400)	13.14 (23/175)			
Gamo Goffa zone	1.48 (6/405)	10.61 (7/66)			
Silte zone	1.95 (8/411)	8.33 (4/48)			
Sheka zone	2.31(8/346)				
Kambata Tembaro zone	2.24 (9/401)				
**All survey sites**			0.034	0.034	< 0.001

*The survey sites were areas where we conducted the two surveys in the same and different health institutions.

^†^R^2^-coefficient of determination weighed for the number of study participants

^‡^P-value for adjusted R^2 ^HIV = Human immunodeficiency virus, ANC = Antenatal care

In all survey sites, where both surveys were conducted in the same as well as in different health institutions, we found no correlation between the rate of HIV infection among pregnant women and TB patients (R^2 ^= 0.034). Briefly, South Omo zone with the highest TB-HIV co-infection rate did not have higher rate of HIV infection among pregnant women whereas Silte zone that had the lowest rate of TB-HIV co-infection did not have the lowest rate of HIV infection among pregnant women (Table 3).

**Figure 2:**
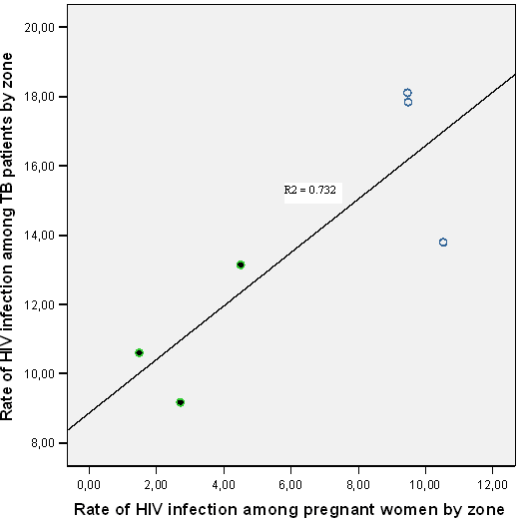
The association of HIV infection among TB patients and pregnant women attending antenatal care in southern Ethiopia, 2004 – 2005.  Urban.  Rural.  Fit line for total.

In contrast, in the six study sites where the two surveys were conducted in the same health institutions, there was a strong correlation between the rate of HIV infection among pregnant women and TB patients (R^2 ^= 0.732). Upon further analysis by residence, the magnitude of correlation was stronger for study participants from urban (R^2 ^= 0.998) than rural areas (R^2 ^= 0.546) as shown in Table 4 and Figure 2. From a linear regression analysis, we found the equation, prevalence of HIV among pregnant women = -6.22 + 0.89* the rate of HIV infection in TB patients. Each per cent increase of HIV seroprevalence in TB patients corresponded to an increase in seroprevalence of 0.89% among pregnant women.

**Table 4:** The rate of HIV infection among TB patients and pregnant women attending antenatal care in the same health institutions of southern region of Ethiopia 2004 – 2005

Survey sites by zones*	**ANC attendants with HIV % (N)**	**TB patients with HIV % (N)**	**R^2†^**	**Adjusted R^2^**	**P – value**
**Urban survey sites**					
Sidama zone	9.48 (29/306)	17.84 (38/213)			
Wolaita zone	10.53 (26/247)	13.79 (12/87)			
Gedeo zone	9.46 (21/222)	18.11 (23/127)			
All urban sites			0.998	0.998	< 0.001
**Rural survey sites**					
Hadiya zone	2.7 (7/259)	9.17 (21/229)			
Gurage zone	4.5 (18/400)	13.14 (23/175)			
Gamo Goffa zone	1.48 (6/405)	10.61 (7/66)			
All rural sites			0.547	0.546	< 0.001
**All survey sites**			0.732	0.732	< 0.001

*The survey sites were areas where we conducted the two surveys in the same health institutions in a district.

^†^R^2^-coefficient of determination weighed for the number of study participants

## Discussion

In the recent decades, the number of TB cases has increased by several folds especially in sub-Saharan African countries. HIV infection is considered the main risk factor for the increase in the number TB patients and the proportion of smear-negative and EPTB cases [3,14,15]. The information about the rate of HIV infection among different groups of a community is important to understand the extent of the problem and to implement appropriate prevention and control measures.

In a large representative survey of TB patients in southern Ethiopia, less than a fifth of them were HIV infected similar to other reports from the region [9,16]. Higher TB-HIV co-infection rates, as high as 47% was reported from Ethiopia [17,18]. These studies however were hospital-based and were conducted in few major towns where the prevalence of HIV infection in the general population was much higher.

In our study, there was no difference in the rate of HIV infection among TB patients by gender, TB classification and category. Unlike several other studies which reported higher rates of HIV infection among smear-negative and EPTB cases compared to smear-positive cases [3,5,10], we did not find difference in the rate of HIV infection among different TB classifications. This could be due to the relatively low prevalence of HIV infection in the region [13]. Another possible explanation could be under diagnosis or referral of some smear-negative and EPTB suspects with a potentially higher risk of HIV infection due to limited diagnostic facilities.

Although the ANC-based HIV sentinel surveillance has weaknesses as the results may be affected by low attendance of ANC, exclusion of private clinics, the rate of contraceptive use and provides no information about men, it has been used as a proxy for HIV prevalence in the general population [9]. In our study, the prevalence of HIV infection among pregnant women attending ANC was 3.8%. This was similar to the previous reports from the region [10] but lower than the reports of sentinel surveillance from other parts of the country [7] and sub-Saharan African countries [19,20]. As expected, the prevalence of HIV among pregnant women was higher in urban areas than rural areas; this could be due to the difference in the risk and rate of HIV infection in urban and rural communities [21,22].

In our study, the rate of HIV infection in TB patients strongly correlated with the rate of HIV infection among pregnant women. This was because HIV is the main risk factor fuelling TB epidemic. Similarly, countries with high HIV prevalence in the general population had higher incidence of TB and relatively higher rates of TB-HIV co-infection.

In southern and eastern Africa, reports have shown an increase in TB notification rate of 13 cases per 10^5 ^population per year for each 1% increase in HIV prevalence in countries with high prevalence of HIV infection [4]. In a generalized HIV epidemic, the rate of HIV infection among TB patients is an indicator of the maturity of the HIV epidemic and predicts the occurrence of new TB cases at country level [9]. A six per cent increase in the number of TB cases and high rates of HIV infection among TB patients over the last two decades were reported from sub-Saharan Africa. This was shown by a strong correlation between adult HIV prevalence and TB case notification in a community; and a higher prevalence of HIV infection in pregnant women was accompanied by high rate of HIV infection in TB patients [19]. Similarly, a strong correlation (R^2 ^= 0.77) was reported from Europe [23].

In our study, the correlation between the seroprevalence in pregnant women and TB patients coincided with the spread and stage of HIV epidemic in a community. This was reflected by the higher rate of HIV infection among TB patients and pregnant women in urban areas. This could be because of matured HIV epidemic in urban areas that led to an increased number of TB cases and number of HIV infected TB patients [24,25]. In rural areas, we found lower correlation possibly due to the low HIV prevalence in the rural communities [13] and a lag period between the spread of HIV infection and maturity of the epidemic. In Zimbabwe, an increase in TB incidence occurred four to five years after the spread of HIV infection in the community [4] and a lag period of seven years was reported from Kenya [26]. Generally, HIV prevalence surveys in Africa, Asia and Pacific showed HIV prevalence in TB patients to be many times higher than that was seen in the general population [27-29]. Similar to the report from Cameroon, surveillance of HIV infection in TB patients could be used as an estimate of the rate of HIV infection in the general population [30].

## Conclusion

The rate of HIV infection in TB patients was associated with the prevalence of HIV infection among pregnant women in the general population. The seroprevalence information for TB patients and pregnant women could be valuable for planning, monitoring and evaluation of joint prevention and control activities. The trend and level of interaction of HIV infection in TB patients and pregnant women need further study.

## Competing interests

The authors declare that they have no competing interests.

## Authors' contributions

DGD, LTC and LEK supervised data collection and laboratory testing. DGD, MAY and BL analysed, interpreted the findings and prepared the drafts. All authors contributed to the final manuscript.

## Pre-publication history

The pre-publication history for this paper can be accessed here:


